# A novel immune-related genes prognosis biomarker for melanoma: associated with tumor microenvironment

**DOI:** 10.18632/aging.103054

**Published:** 2020-04-20

**Authors:** Rongzhi Huang, Min Mao, Yunxin Lu, Qingliang Yu, Liang Liao

**Affiliations:** 1 The First Affiliated Hospital of Guangxi Medical University, Nanning 530021, The Guangxi Zhuang Autonomous Region, China; 2 Department of Traumatic Orthopedics and Hand Surgery, The First Affiliated Hospital of Guangxi Medical University, Nanning 530021, The Guangxi Zhuang Autonomous Region, China

**Keywords:** melanoma, immune-related genes, classifier, overall survival, microenvironment

## Abstract

Background: Melanoma is a cancer of the skin with potential to spread to other organs and is responsible for most deaths due to skin cancer. It is imperative to identify immune biomarkers for early melanoma diagnosis and treatment.

Results: 63 immune-related genes of the total 1039 unique IRGs retrieved were associated with overall survival of melanoma. A multi-IRGs classifier constructed using eight IRGs showed a powerful predictive ability. The classifier had better predictive power compared with the current clinical data. GSEA analysis showed multiple signaling differences between high and low risk score group. Furthermore, biomarker was associated with multiple immune cells and immune infiltration in tumor microenvironment.

Conclusions: The immune-related genes prognosis biomarker is an effective potential prognostic classifier in the immunotherapies and surveillance of melanoma.

Methods: Melanoma samples of genes were retrieved from TCGA and GEO databases while the immune-related genes (IRGs) were retrieved from the ImmPort database. WGCNA, Cox regression analysis and LASSO analysis were used to classify melanoma prognosis. ESTIMATE and CIBERSORT algorithms were used to explore the relationship between risk score and tumor immune microenvironment. GSEA analysis was performed to explore the biological signaling pathway.

## INTRODUCTION

Melanoma is a life-threatening malignancy with high metastasis and mortality rates [1, 2]. Approximately 232,000 new melanoma patients were diagnosed in 2011 and with 55,000 deaths recorded in the same year [3]. High mortality rates result from poor prognosis leading to late diagnosis. Therefore, there is need to come up with approaches for early diagnosis [4–6].

The TNM stage is an effective approach for detection of the cancer stage, is invaluable in cancer prognosis and informs on the right therapy approaches [7]. However, differences in the overall survival associated with TNM stage method are observed [8]. Current studies on tumors have revealed the clinical limitations of TNM stage method [9, 10]. Therefore, there is a need to explore new melanoma markers to guide the clinical treatment and improve melanoma prognosis. Gene-based biomarkers have become more popular with the advances in human gene sequencing [11, 12].

Most immune system components are implicated in the initiation and progression of melanoma [13, 14]. In tumor immunity, tumor cells act as antigens while immune cells and leukocytes infiltrates the tumor tissue function through chemotaxis for immune defense [13]. Immune escape also is an important factor in tumorigenesis [15, 16]. Currently, a myriad of new immunotherapy are used in melanoma and including PD-1, PD-L1 and CTLA-4 inhibitors [17, 18]. However, these approaches are effective only on a few patients while the majority of the patients have limited or no response to the therapy especially during melanoma progression [19, 20]. Therefore, comprehensive analyses of the correlation between immune genes and overall survival in melanoma are important in exploring the potential prognostic value of immune genes and new biomarkers.

In this study, our aim was to construct a novel immune-related genes biomarker for use in immunotherapies and melanoma prognosis. Comprehensive bioinformatics analyses were performed to explore underlying mechanisms of the biomarker. This study provides information for subsequent personalized diagnosis and treatment of melanoma.

## RESULTS

### Identification of survival-related modules by WGCNA

**Figure 1:**
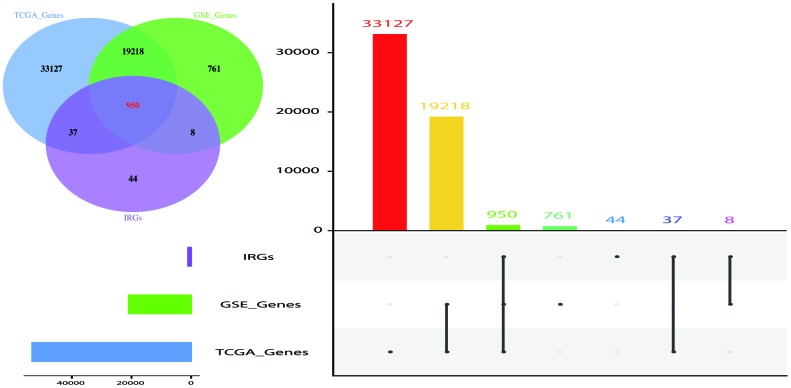
**Venn diagram and Histogram was used to visualize common IRGs shared between GEO dataset, TCGA dataset and IRGS.** 950 IRGs overlapped in the three datasets. The value used represented the number of gene symbol covered from the ensemble IDs and probe IDs. The number of genes annotated are presented on the y-axis.

**Figure 2:**
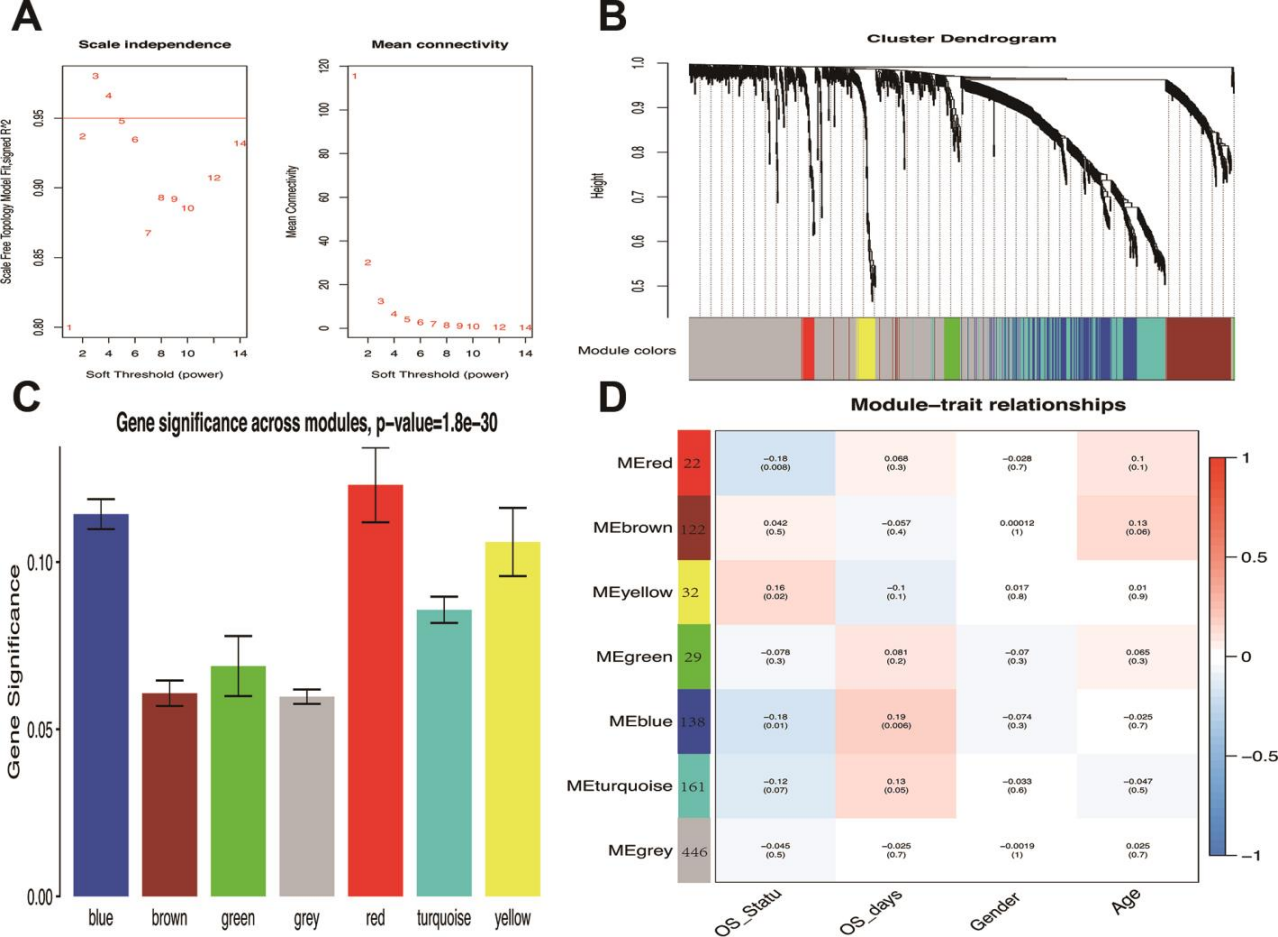
**Weighted melanoma gene co-expression network.** (**A**) The scale-free fit index for soft-thresholding powers. The soft-thresholding power in the WGCNA was determined based on a scale-free R2 (R2 = 0.95). The left panel presents the relationship between the soft-threshold and scale-free R2. The right panel presents the relationship between the soft-threshold and mean connectivity. (**B**) A dendrogram of the differentially expressed genes clustered based on different metrics. Each branch in the figure represents one gene, and every color below represents one co-expression module. (**C**) Distribution of average gene significance and errors in the modules associated with overall survival of melanoma patients. Based on the average linkage hierarchical clustering and the soft-thresholding power, six modules were identified. To determine the significance of each module, gene significance (GS) was calculated to measure the correlation between genes and sample traits. GS was defined as the log10 conversion of the p-value in the linear regression between gene expression and clinical data (GS = lg P). The red and blue module showed high correlation with the survival of melanoma patients. (**D**) A heatmap showing the correlation between the gene module and clinical traits. The red module contained 22 IRGs while the blue module contained 138 IRGs. The correlation coefficient in each cell represented the correlation between gene module and the clinical traits, which decreased in size from red to blue. The blue module showed the highest positive correlation with the survival while the red module showed the highest negative correlation with the survival.

WGCNA analysis was carried out on 950 overlapping IRGs (Figure 1). The soft-thresholding power in WGCNA was determined based on a scale-free R2 (R2 = 0.95). Six modules were identified based on the average linkage hierarchical clustering and the soft-thresholding power. The red module showed the highest correlation with the overall survival of melanoma. Additionally, the blue module was highly correlated with the overall survival of melanoma. The red module contained 22 IRGs while the blue module contained 138 IRGs (Figure 2). Data for these two modules were selected for further analysis.

### Construction of prognostic classifier based on IRGs

**Figure 3:**
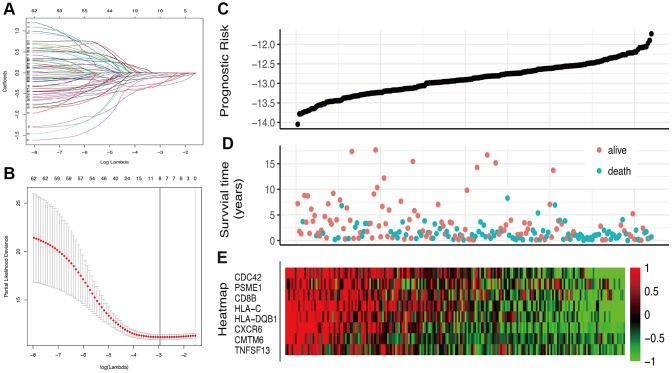
**Construction of the IRGs prognostic classifier.** (**A**, **B**) Determination of the number of factors by the LASSO analysis. (**C**) The distribution of RS. (**D**) The survival duration and status of patients. (**E**) A heatmap of IRGs in the classifier.

**Figure 4:**
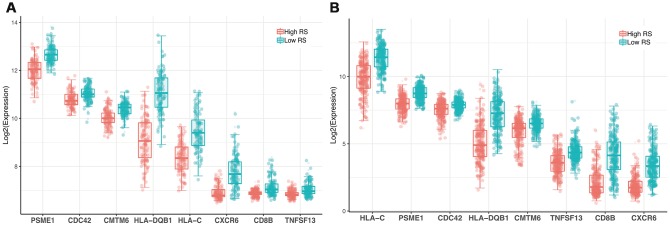
**Expression profile of 8 genes.** (**A**) GSE dataset (**B**) TCGA dataset.

**Figure 5:**
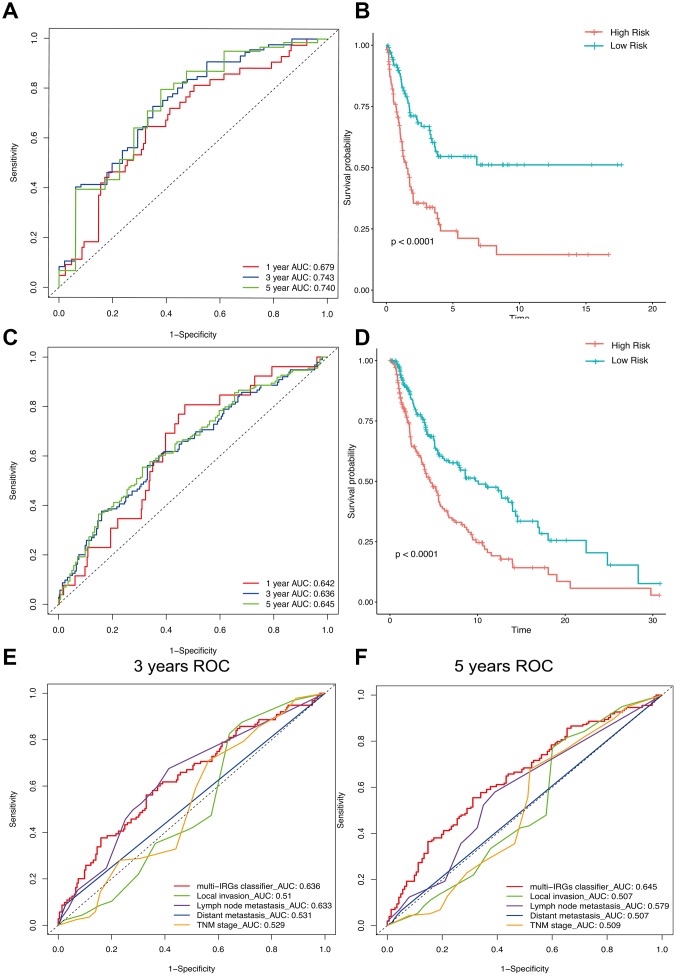
**The distribution of time-dependent ROC curves and Kaplan-Meier survival based on the integrated classifier in the training and independent validation sets.** ROC, receiver operator characteristic. AUC, the area under the curve. (**A**) ROC curve for the GSE cohort. (**B**) KM curve of the GSE cohort. (**C**) ROC curve of the TCGA cohort. (**D**) KM curve of the TCGA cohort. (**E**) 3-years correlation ROC curve in the TCGA cohort for the comparison of the classifier prognostic accuracy and clinical characteristics. (**F**) 5-years correlation ROC curve in the TCGA cohort for the comparison of the classifier prognostic accuracy and clinical characteristics.

63 IRGs of the red and blue modules were identified as survival related IRGs of melanoma with the criterion of P < 0.01 (Supplementary File 1). LASSO analysis identified eight IRGs (PSME1, CDC42, CMTM6, HLA-DQB1, HLA-C, CXCR6, CD8B, TNFSF13) which were included in the classifier (Figure 3). The coefficients of the eight IRGs are shown in Table 1 and the expression levels are shown in Figure 4. The high-RS group showed a poor overall survival rate compared with low-RS group based the Kaplan-Meier analysis (Figure 5B). Time-dependent ROC curves showed that the classifier had a strong predictive ability in GSE dataset (Figure 5A). In the training cohort, the AUC was 0.679 in 1 year, 0.743 in 3 years and 0.740 in 5 years (Figure 5A).

**Table 1:** The IRGs in the prognostic classifier associated with OS in the GSE dataset.

**Symbol**	**Univariate Cox regression analysis**			**LASSO coefficient**
	**HR**	**95%CI**	**P Value**	
PSME1	0.416	0.285-0.608	5.854205e-06	-0.30396287
CDC42	0.428	0.248-0.74	0.00236537	-0.24399092
CMTM6	0.364	0.218-0.608	0.0001131757	-0.23548175
HLA-DQB1	0.692	0.592-0.809	3.711835e-06	-0.07311844
HLA-C	0.595	0.466-0.759	2.920363e-05	-0.10691953
CXCR6	0.509	0.363-0.713	8.635839e-05	-0.03143482
CD8B	0.248	0.108-0.566	0.0009273984	-0.05032655
TNFSF13	0.172	0.055-0.54	0.002576346	-0.25872281

### Verification of the prognostic classifier in TCGA cohort

We used the TGCA cohort to validate the predictive ability of the classifier. Kaplan-Meier analysis showed that the high-RS group had a poor overall survival (P<0.0001, Figure 5D). Time-dependent ROC curves showed that the classifier had a good accuracy with 0.642 in 1 year, 0.636 in 3 years and 0.645 in 5 years (Figure 5C). Moreover, the classifier had better predictive power and accuracy compared with other clinical features (Figure 5E, 5F). In Addition, the classifier was an independent factor in multivariate Cox analysis. Results of univariate and multivariate analyses in prognostic factors and overall survival were showed in Table 2.

**Table 2:** Univariate and multivariate analyses of prognostic factors and overall survival of melanoma patients in TCGA cohort.

**Characteristics**	**Univariate Cox regression analysis**			**multivariate Cox regression analysis**		
	**HR**	**95%CI**	**P Value**	**HR**	**95%CI**	**P Value**
Age	1.025	1.015-1.035	3.63e-07	1.021	0.01-4.06	4.83e-05
Gender	0.877	0.655-1.175	3.79e-01	1.088	0.16-0.54	0.592
Local invasion	0.988	0.955-1.021	4.65e-01	0.987	0.02--0.66	0.511
Lymph node metastasis	1.087	1.032-1.145	1.74e-03	1.092	0.03-3.3	0.00096
Distant metastasis	1.161	0.887-1.52	2.78e-01	1.429	0.14-2.55	0.0107
TNM stage	1.000	0.964-1.038	9.80e-01	0.982	0.02--0.75	0.455
Multi-IRGs Classify	1.588	1.315-1.919	1.61e-06	1.704	0.1-5.13	2.94e-07

### Immune infiltration score between high and low RS group

**Figure 6:**
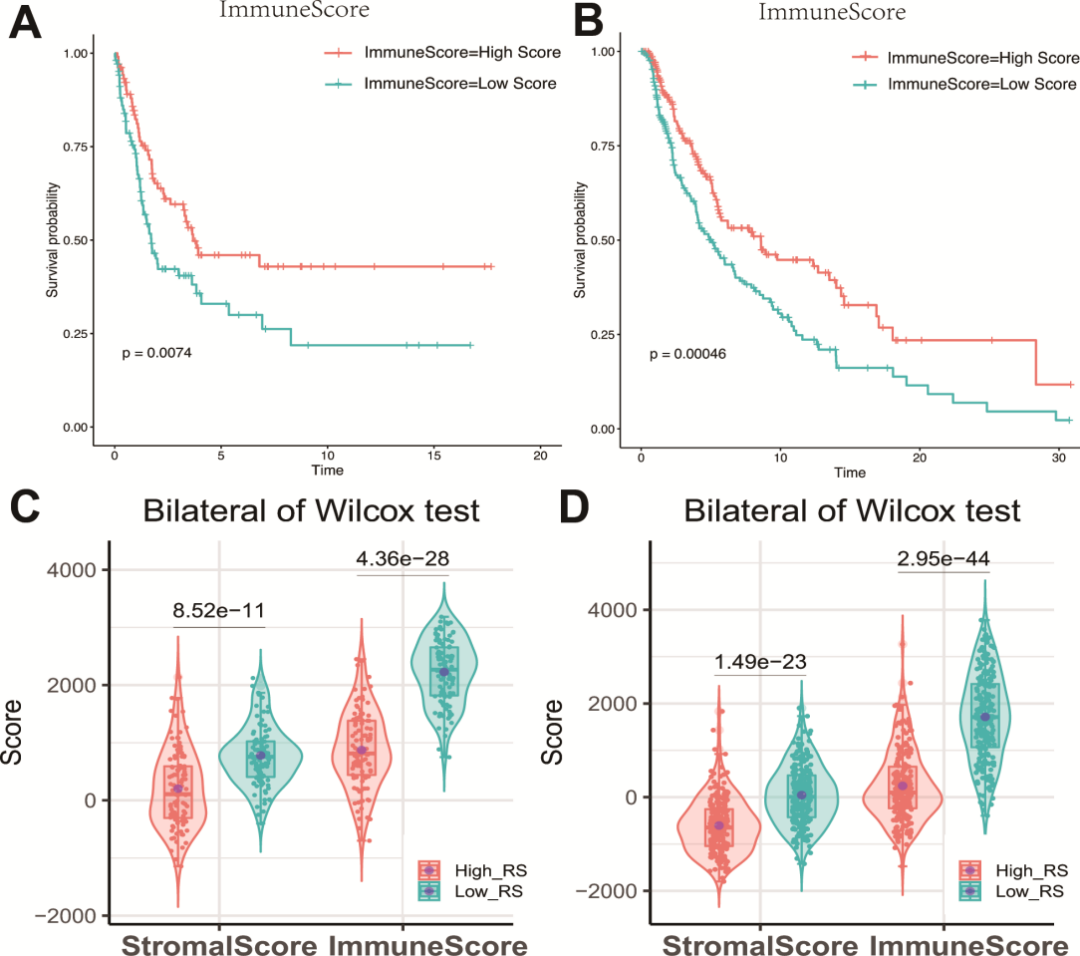
(**A**) Impact of immune score on overall survival in melanoma based on KM analysis. (**A**) GSE cohort. (**B**) TCGA cohort. (**C**, **D**) Association with immune score, stromal score and risk score. The high-RS group showed lower immune score and stromal score comparing with low-RS group. (**C**) GSE cohort. (**D**) TCGA cohort.

Kaplan-Meier analysis showed that different immune scores had differential overall survival in melanoma samples (Figure 6A, 6B). The immune score showed a significant difference between high and low-RS group (Figure 6C, 6D).

### Immune cell subtypes between high and low RS group

**Figure 7:**
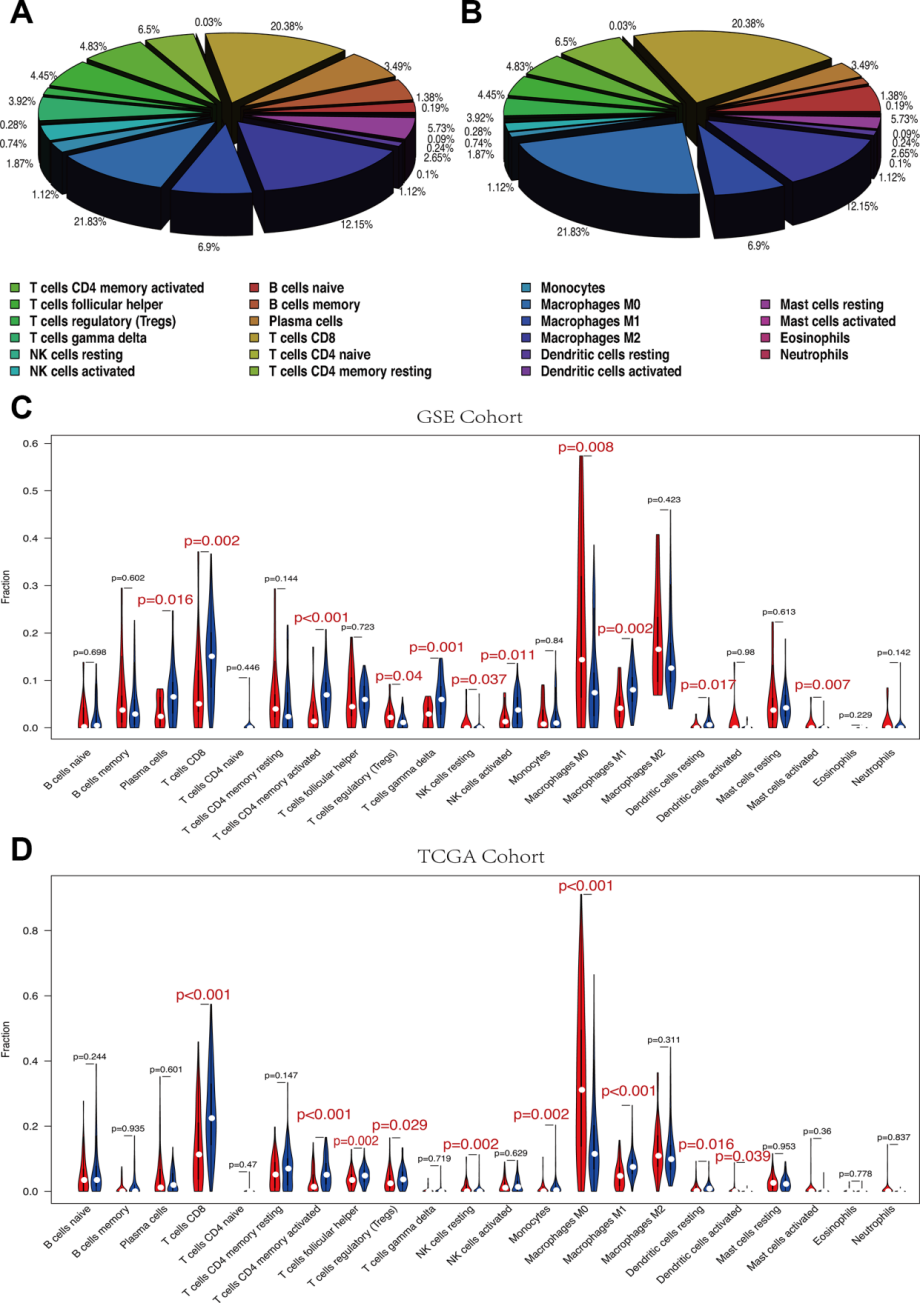
(**A**, **B**) The mean proportion of 22 immune cells in GSE cohort. Macrophages M0, Macrophages M2 and T cells CD8 account for a large proportion of melanoma immune cell infiltration. (**A**) GSE cohort. (**B**) TCGA cohort. (**C**, **D**) Violin plot showing the relationship between risk score with immune score and stromal score. Red color represents high-RS group while blue color represents low-RS group. Differential immune cell type expression was observed between the high and low-RS groups. (**C**) GSE cohort. (**D**) TCGA cohort.

The 22 immune cell proportions of melanoma are shown in Figure 7A, 7B. Macrophages M0, Macrophages M2 and T cells CD8 accounted for a large proportion of melanoma immune cell infiltration. High and low RS groups showed differential immune cells expression (Figure 7C, 7D).

### GSEA analysis

**Figure 8:**
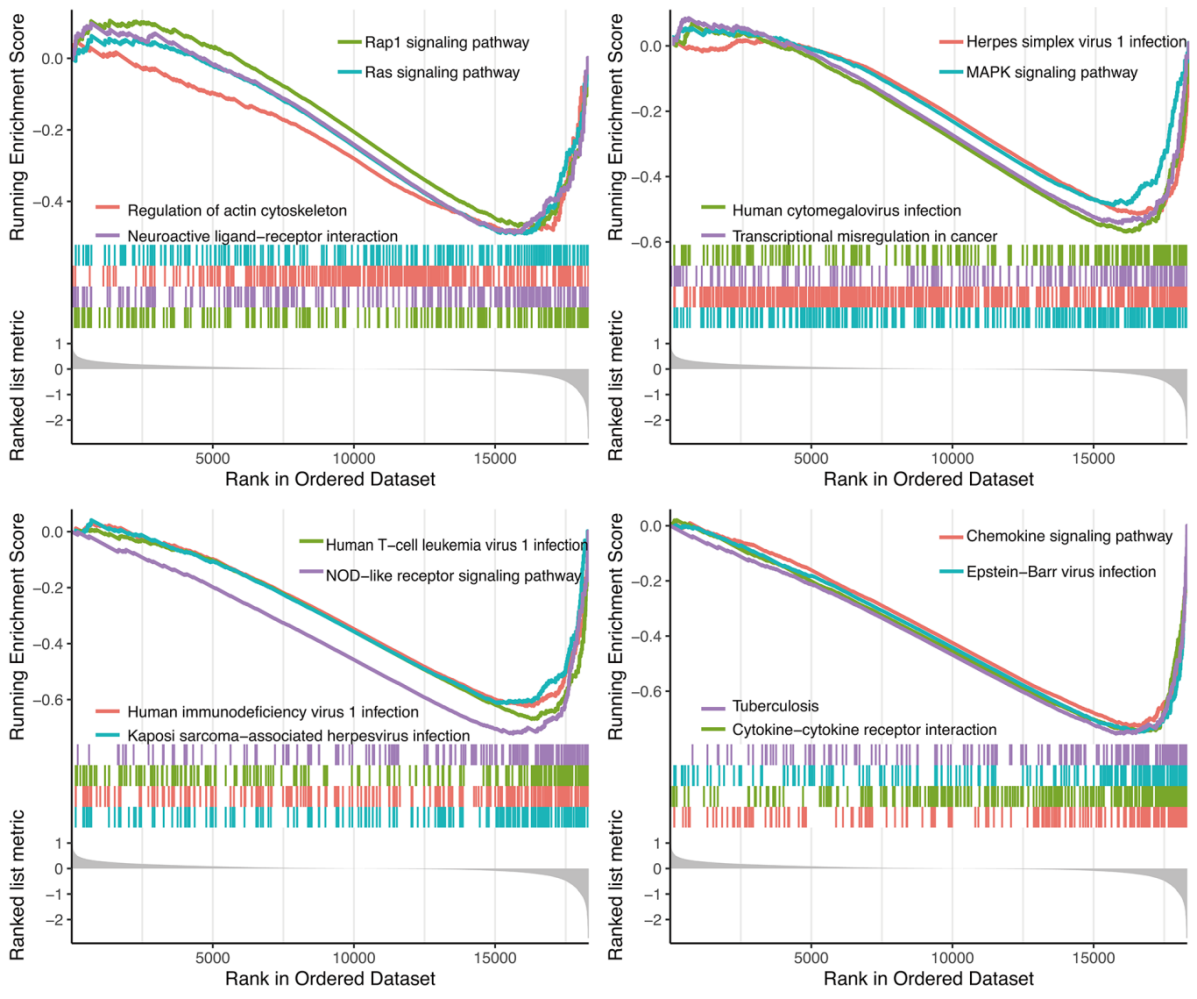
**GSEA analysis.**

GSEA analysis showed 14 significant KEGG pathways associated with risk score, including Rap1 signaling pathway, Ras signaling pathway, Herpes simplex virus 1 infection, Regulation of actin cytoskeleton, MAPK signaling pathway, Neuroactive ligand-receptor interaction, Human cytomegalovirus infection, Human T-cell leukemia virus 1 infection, Human immunodeficiency virus 1 infection, Kaposi sarcoma-associated herpesvirus infection, Chemokine signaling pathway, Epstein-Barr virus infection, Tuberculosis and Cytokine-cytokine receptor interaction (Figure 8).

## DISCUSSION

Melanoma is a fatal skin cancer that affects many people worldwide each year [21]. Currently, immunotherapy is a successful treatment option for melanoma [22]. Notably, many researchers demonstrates the role of the immune cells on tumor cells [23, 24]. Moreover, immune components in melanoma tissue can be used to evaluate therapeutic efficacy and melanoma prognosis in patients [25]. In this study, 63 IRGs were found to be associated with melanoma prognosis, of which eight IRGs were adopted to construct a classifier. The classifier showed reliable predictive value and accuracy. In addition, we explored the relationship between RS and the prognosis value in melanoma. The findings showed differences in immune cell infiltration and multiple signaling pathways between high and low-RS group.

The PSME1, CDC42, CMTM6, HLA-DQB1, HLA-C, CXCR6, CD8B and TNFSF13 RGs were used in the classifier. These IRGs were reported to be associated with tumor prognosis in previous studies. Cell division cycle 42 (CDC42) protein, a member of Rho GTPases, activates multiple cellular processes by regulating actin cytoskeleton [26]. In addition, CDC42 facilitates the invasion and migration of melanoma cells [27–29]. Therefore, CDC42 inhibitors have been effective in melanoma treatment [30, 31]. CMTM6 is a ubiquitously expressed protein encoded by two distinct gene clusters located on chromosome 16 and chromosome 3 [32]. It enhances PD-L1 expression and anti-tumor immunity. Therefore, CMTM6 is a potential biomarker and therapeutic target for melanoma patients [33–35]. Among the HLA class I antigens, HLA-C locus recognizes the inhibitory killer cells and suppresses the functions of NK cells in melanoma patients [36–38]. Furthermore, the frequency of HLA-DQB1*0301 and HLA-DQB1*0303 alleles are highly expressed in melanoma patients [39]. Moreover, melanoma patients with DQBI*0301 allele have thicker primary tumor and are more likely to have local or distant metastatic disease [40]. Besides, the chemokine co-receptor CXCR6 was identified as a new biomarker associated with asymmetric self-renewal of tissue-specific stem cells. CXCR6 + cells cause rapid increase in tumor mass compared with CXCR6- cells [41]. TNFSF13, a member of the TNF superfamily, was reported to indicate the proliferative or survival state in tumor cells [42]. The multi-IRGs classifier established in this study showed high predictive value and accuracy through various analyses.

The degree of immune infiltration significantly affected melanoma survival. Previous studies demonstrate that immune cells in the tumor microenvironment can be used in the prognostic assessment of multiple tumors, such as glioblastoma, breast cancer, and melanoma [43–45]. In this study, the expression of eight genes affected immune infiltration scores. Patients with higher immune scores had better prognosis. This finding implies that prognosis value of risk score is associated with melanoma immune system.

To further explore the immune and risk score, we used the CIBERSORT algorithms to calculate the immune cell subtype in R platform. Our result showed that the two risk score groups expressed differential immune cell subtypes. Ali et al. demonstrated that imbalance in immune cell component ratio is highly correlated with poor prognosis and low survival in cancer patients [46, 47]. A previous study reported that CD8^+^ T cells produces granulocyte and perforin to kill tumor cells [48]. In our study, the immune cells found in melanoma mainly comprised macrophages M0, macrophages M2 and T cells CD8. In this study, T cell CD8 levels were low whereas M0 and M2 macrophages levels were high in the high-risk group. This implies that imbalance of T cell CD8 and M0, M2 macrophage ratio may reduce the survival rate of patients in the high-risk group. High expression of CD8+T cells may improve the prognosis of melanoma patients as well as reduce the risk factors.

GSEA analysis showed differences in 14 important signaling pathways between high and low RS groups. Inhibition of MAPK signaling pathway improved melanoma immune microenvironment by enhancing the melanoma antigen expression and down-regulating immunosuppressive cytokines [49, 50]. Additionally, chemokine signaling pathway participates in tumor growth. Some chemokines, such as CCR10 and CXCR3, have been shown to play an important role in the proliferation and metastasis of melanoma cells [51, 52].

In this study, LASSO regression analysis was used to establish a novel classifier using multiple IRGs and the classifier was verified using an independent cohort. Currently, few studies have used ESTIMATE and CIBERSORT algorithms to explore immune infiltration in melanoma. In this study, we use these algorithms to explore immune infiltration in melanoma using the R software. These preliminary results could provide a perspective for exploring the role of immune infiltration in melanoma. However, this study has the following limitations. First, the reliability of our molecular mechanism analysis results is limited due to lack of vitro or vivo experiments. Second, this study was a retrospective study, therefore, prospective study should be carried out to validate the findings of our study.

In conclusion, we successfully constructed a multi-IRGs classifier with the powerful predictive function. Differences in the overall survival of high and low risk groups are implicated in immune infiltration, tumor microenvironment and the interaction of multiple signaling pathways. This study provides additional information on the analysis of melanoma pathogenesis and clinical treatment.

## MATERIALS AND METHODS

### Data Procession

GSE65904 gene expression profiles were retrieved from the gene expression omnibus database (GEO: https://www.ncbi.nlm.nih.gov/geo/). In this study, the samples with no follow-up information or follow-up time less than 1 day were excluded. 210 melanoma samples were retrieved for subsequent analysis. Further, the probe IDs were converted to gene symbols using the illuminaHumanv4.db R package. The probe IDs with the highest mean value were reversed when more than one probe had a matched gene symbol. The GEO expression file was converted into log2 (expression) for further analysis. Additionally, the RNA-FPKM data and clinical data of melanoma samples were retrieved for external validation analysis using the TCGA biolinks R package. Samples with no follow-up information or follow-up time less than 1 day were excluded. The expression file of patients with the highest mean value was reversed when more than one expression file had matched patients. 428 melanoma samples in TCGA were used for analysis.

### Immune-related gene extraction

Immune-related genes (IRGs) data were retrieved from the ImmPort database (https://immport.niaid.nih.gov) (Supplementary File 2). Overlapping immune-related genes from the GEO dataset, TCGA dataset and IRGs were selected for further analysis.

### Weighted gene co-expression network analysis

GEO expression file was used for weighted gene co-expression network analysis (WGCNA) using WGCNAR package. WGCNA was used to explore the relationship between the clinical features with expression modules. Module eigengenes (MEs) were defined as the first principal component of each gene module and adopted as the representative of all genes in each module. Gene significance (GS), as the mediator p-value (GS = lg P) for each gene, represented the degree of linear correlation between gene expression of the module and clinical features. Survival-related modules were defined according to P≤0.01 and the higher GS value was extracted for further analysis.

### LASSO analysis

Univariate Cox regression analysis was performed to explore the impact of each gene on overall survival. The IRGs of survival-related modules with P<0.01 were identified as survival-related IRGs and integrated into the Least Absolute Shrinkage and Selection Operator (LASSO) regression for identification of prognostic risk signatures. The risk score (RS) of each sample was calculated using the formula: risk score = Σexpgenei* βi.

The Kaplan-Meier curve analysis was further conducted to evaluate the relationship between the risk score and overall survival. The median value was used as the cut-off. Univariate and multivariate Cox regression analysis were performed to study the relationship between the index and the clinical features. To validate the accuracy and predictive ability of the signature, it was included in the TCGA dataset. The area under the curve (AUC) of the ROC curve was calculated and compared to examine the classifier performance using time ROC R package.

### Comparison of the degree of immune cell infiltration between high and low RS groups

To explore the relationship between risk score and melanoma prognosis, we analyzed the relationship between risk score and tumor microenvironment. The tumor microenvironment comprises a variety of cell types, including immune cells, mesenchymal cells, endothelial cells, inflammatory mediators, and extracellular matrix (ECM) molecules [53]. We used the ESTIMATE algorithm to determine the immune score of each sample using R software and further compared the difference in degree of immune cell infiltration between high and low-risk groups by Wilcoxon test.

### Comparison of 22 immune cell subtypes between high RS and low RS groups

To explore the differences of immune cell subtypes, CIBERSORT package was used to assess the proportions of 22 immune cell subtypes based on expression file [54]. The perm was set at 1000. Samples with P < 0.05 in CIBERSORT analysis result were used in further analysis. Mann-Whitney U test was used to compare differences in immune cell subtypes in the high RS and low RS groups.

### Gene Set Enrichment analysis (GSEA)

To identify signaling pathway that are differentially activated between the high RS and low RS groups, we selected an ordered list of genes through limma R package and conducted Gene Set Enrichment Analysis (GSEA) with adjusted p < 0.05 using the cluster filer R package.

### Statistical analysis

All analyses were carried out by R version 3.5.2 and corresponding packages. Kaplan-Meier analysis was further conducted to evaluate the relationship between immune score and overall survival using the survimer R package. The median value was set as the cut-off. The glmnet R package was used for LASSO analysis.

### Availability of data and materials

The GSE65904gene expression profiles were retrieved from GEO (https://www.ncbi.nlm.nih.gov/geo). The TCGA data were retrieved from GDC data portal (https://portal.gdc.cancer.gov/). The immune-related genes (IRGs) data were retrieved from the ImmPort database (https://immport.niaid.nih.gov). The R software (https://www.r-project.org/) was used for all statistical analyses.

## Supplementary Material

Supplementary File 1

Supplementary File 2
